# Learning to Produce Syllabic Speech Sounds via Reward-Modulated Neural Plasticity

**DOI:** 10.1371/journal.pone.0145096

**Published:** 2016-01-25

**Authors:** Anne S. Warlaumont, Megan K. Finnegan

**Affiliations:** 1 Cognitive and Information Sciences, University of California, Merced, Merced, CA, United States of America; 2 Speech & Hearing Sciences, University of Illinois at Urbana-Champaign, Champaign, IL, United States of America; Ghent University, BELGIUM

## Abstract

At around 7 months of age, human infants begin to reliably produce well-formed syllables containing both consonants and vowels, a behavior called canonical babbling. Over subsequent months, the frequency of canonical babbling continues to increase. How the infant’s nervous system supports the acquisition of this ability is unknown. Here we present a computational model that combines a spiking neural network, reinforcement-modulated spike-timing-dependent plasticity, and a human-like vocal tract to simulate the acquisition of canonical babbling. Like human infants, the model’s frequency of canonical babbling gradually increases. The model is rewarded when it produces a sound that is more auditorily salient than sounds it has previously produced. This is consistent with data from human infants indicating that contingent adult responses shape infant behavior and with data from deaf and tracheostomized infants indicating that hearing, including hearing one’s own vocalizations, is critical for canonical babbling development. Reward receipt increases the level of dopamine in the neural network. The neural network contains a reservoir with recurrent connections and two motor neuron groups, one agonist and one antagonist, which control the masseter and orbicularis oris muscles, promoting or inhibiting mouth closure. The model learns to increase the number of salient, syllabic sounds it produces by adjusting the base level of muscle activation and increasing their range of activity. Our results support the possibility that through dopamine-modulated spike-timing-dependent plasticity, the motor cortex learns to harness its natural oscillations in activity in order to produce syllabic sounds. It thus suggests that learning to produce rhythmic mouth movements for speech production may be supported by general cortical learning mechanisms. The model makes several testable predictions and has implications for our understanding not only of how syllabic vocalizations develop in infancy but also for our understanding of how they may have evolved.

## Introduction

### Emergence of syllabic babbling in humans

In the first year of life, infants undergo a transition from non-syllabic babbling to syllabic babbling. Syllables that have both a well-formed consonant and a well-formed vowel, with adult-like timing of the transition between the consonant and vowel, are called canonical syllables. The precursors to canonical babbling, in the form of primitive tongue or lip movements often referred to as “gooing” and then “marginal babbling”, are present from around 2–3 months of age. Over the next few months, the consonants and vowels the infants produce come to be more clearly articulated. True canonical syllables typically appear consistently in a child’s repertoire at about 7 months of age and continue to increase in frequency relative to non-canonical vocalizations over the next several months [1–4]. Canonical babbling development forms a critical foundation for human speech. The specific consonant and vowel sounds present in an infant’s prelinguistic canonical babbling tend to be the same sounds that are present in the infant’s first words [5]. The milestone of consistent production of canonical syllables has been shown to be a salient event for parents [6].

From behavioral studies, it appears that learning plays a critical role in the development of canonical babbling in human infancy. The fact that canonical babbling emerges gradually over the course of several months, rather than being present at birth, suggests the possibility that it is a learned behavior, although a protracted course of development does not in and of itself strongly indicate a learned basis (it is possible that protracted development could arise from physical maturation not involving learning). More convincing evidence for the role of learning in canonical babbling comes from the fact that infants with severe or profound hearing impairment but who are otherwise typically developing exhibit significant delays in canonical babbling onset [7–9] and produce fewer consonants per utterance [10]. Furthermore, the age of onset of canonical babbling correlates positively with the age of cochlear implantation [11]. These findings suggest that audition plays a major role in the development of canonical babbling and are consistent with the idea that auditory stimulation reinforces infants vocal motor learning in favor of syllabic sounds.

Additionally, a case study of an infant who was tracheostomized from 5–20 months of age found that when decannulated at 20 months, the child’s pattern of babbling resembled that of an infant 6 months of age or younger, in that very few utterances contained canonical syllables and the child had a rather small consonant repertoire within those canonical utterances [12, 13]. In many ways, the infant’s vocalization pattern resembled that of profoundly deaf infants. Tracheostomy does not prohibit the infant from moving the upper vocal tract in ways that would lead to syllable production (e.g., the lips and tongue can move freely), but it does make these vocalizations soundless (except when the individual blocks the flow of air from the cannula), so that the auditory consequences of moving the lips and jaw while phonating are not experienced by the infant. A reasonable conclusion is thus that experience producing vocalizations, and in particular learning about the auditory consequences of vocal tract movements, is necessary for the development of canonical, i.e. syllabic, babbling [12, 14].

### Possible neural mechanisms underlying the development of syllabic speech

Humans are the only primate species that produces canonical babbling. While nonhuman primates do not produce syllabic vocalizations containing canonical consonants and vowels, they do produce rhythmic orofacial movements during chewing and sucking for feeding purposes. It has been proposed that these feeding movements, especially chewing, were an evolutionary precursor to human syllabic speech, and that in humans speech and chewing have a common neural basis. Specifically, MacNeilage [15] has proposed that the evolutionary trajectory may have been one of transition from mandibular oscillation for ingestion to mandibular oscillation in lipsmacking, and then to mandibular oscillation in human speech.

Lipsmacks are a type of communicative signal used by a number of different species of primates, such as a macaques, baboons, and chimpanzees, usually during face-to-face social interactions. They typically occur in the absence of phonation (i.e. sound production at the larynx) and so are sometimes referred to as facial expressions rather than vocalizations, although in some species lipsmacks have been reported to occur superimposed upon phonation. They show some very striking similarities to human syllable production. The rate of mandibular oscillation in lipsmacking in rhesus macaques is roughly 5 Hz, which is very similar to the rate of syllable production in adult speech. Furthermore, infant monkeys have slower rhythms in their lipsmacking, with the rate gradually ramping up to the 5 Hz rate seen in adult lipsmacks [16]. Similarly, when human infants first begin babbling, the rate of syllable production is typically considerably slower than the rate of syllable production in adult speech. Lipsmacking may therefore be an evolutionary precursor to the upper vocal tract movement component of syllabic human speech production [16].

If it is true that syllabic speech originates from primate lipsmacking, and perhaps that both originated from ingestive behaviors, then human speech may recruit existing central pattern generators for rhythmic oral movement located in the brainstem [15, 17]. It has been argued that at the age when canonical babbling is emerging, human infants utilize “phyologenetially old neuromuscular coordinations” [18], since they do not yet have mature voluntary cortical control of movement. If this is the case, the details of how these circuits get recruited are largely unknown.

Another possibility is that the ontogenetic development of syllabic vocal babbling is largely due to learning in motor regions of the neocortex. It has been found that direct stimulation of the supplementary motor area in adult humans can, at least in some cases, elicit reduplicated babbling sequences such as repetitions of the syllable “da” or “te” [15, 19, 20]. Patients with paroxysmal lesions to the same region have also been reported to exhibit these types of syllable repetitions [21]. Others have found in macaques that stimulation of the precentral motor cortex and related regions can generate rhythmic jaw movement [22]. There is therefore ample evidence that there are regions of the posterior frontal lobe that, when stimulated, lead to rhythmic speech and/or jaw movement. It seems likely then that reduplicated babbling relies in some way on cortical mechanisms.

Involvement of motor regions of the cortex in production of syllabic vocalizations does not in and of itself necessarily imply that the cortex is doing fine-grained programming of vocal tract movements for speech. It is possible that the role played by these regions of motor cortex is to recruit brainstem circuits, and that the brainstem circuits perform the fine-grained programming of the movements. However, recent findings indicate that the temporal dynamics of cortical activity can indeed be mapped quite closely to temporal dynamics of articulator movements [23]. Furthermore, direct stimulation of specific regions of the precentral motor cortex elicits vocal fairly specific movements of parts of the vocal tract, such as movements of the vocal folds, movements of the lips, and movements of the jaw, in primates [22, 24, 25], allowing for the possibility that the motor cortex is at least capable programming of vocal tract movements for speech quite directly and in a detailed manner.

Given the strong evidence that canonical babbling requires some learning and is not merely the result of purely maturational processes sans learning, and given the large degree to which learning is known to be involved in the development of cortical circuits, it is worth exploring the possibility that cortical learning plays a role in the development of speech sound production. During the first year of life, regions of the motor cortex may acquire the ability to directly generate movements of the vocal tract articulators that result in syllabic vocalizations.

That the development of syllabic babbling relies on cortical learning is consistent with the idea that domain-general learning mechanisms underlie early speech sound acquisition [26]. It is also consistent with the idea that more elaborate cortical circuitry for coordinated control of phonatory movements in humans compared to other primates is related to humans’ vocal learning abilities, and subsequently their capacity for speech [15, 24, 25].

The present paper describes a computational model that supports the idea that cortical learning within the motor cortex could indeed lead to generation of syllabic vocalizations.

### Our modeling approach

The model presented here combines a spiking neural network with a realistic model of the human vocal tract. Oscillations of cortical neurons in our model are shown to be capable of driving muscles that lead to sounds some of which are more auditorily salient than others. Reinforcement is correlated with the production of canonical syllables and triggers dopamine-modulated spike-timing-dependent plasticity (DA-modulated STDP), yielding learning. The model relies on neurophysiologically realistic mechanisms. Canonical babbling development is exhibited. For simplicity, we focus on lip and jaw movement (treated together as a single motor degree of freedom). Oscillatory movements of these structures are associated with infants’ bilabial reduplicated babbling sounds, such as the sequence /bababa/ [27].

It is reasonable to assume that caregivers prefer, or at least are more attentive to, more salient sounds as opposed to less salient sounds. Indeed, observation of naturalistic mother-infant interactions has shown that infant utterances containing both consonants and vowels are more likely to receive interactive vocal responses from mothers than infant utterances that contain only vowel elements [28]. Other work has shown that adults prefer infant vocalizations that are longer, less nasal, and contain intonational contours [29–31], all features that might be expected to correlate with auditory salience [32, 33]. These social responses are presumably rewarding to the infant in and of themselves, and they are likely correlated with provision of food and other resources that have rewarding value to infants [26]. It is also reasonable to assume that infants are more stimulated by their own vocalizations when those self-generated stimuli are more salient, although no behavioral studies have yet tested this idea (we will return to it in the Discussion when we discuss the testable predictions made by our model). It has been shown that infants prefer caregiverese to adult-directed speech [34], and that this preference appears to be driven by the salient frequency modulations in caregiverese [35]. Auditory salience can be estimated automatically [36, 37], and tends to be higher when vocalizations contain both consonants and vowels than when vocalizations contain only vowels [38, 39]. In the model presented here, reinforcement is based on auditory salience (see also [40, 41]).

The present modeling approach contrasts with that of most other computational models of infant vocal learning in that it focuses on the neural basis of the emergence of syllabically structured vocalizations in the infant’s vocal repertoire [42]. Many models of vocal learning focus solely on vowel production [43–50]. Focusing only on vowel learning allows the modeler to avoid addressing the temporal dynamics of movement, since vowels can be reasonably characterized by and synthesized given a single, static configuration of the vocal tract articulators. For testing general principles of sensorimotor mapping, exploratory strategies, the role of imitation, etc. applied to an aspect of speech learning, this simplification has been helpful. However, it is clear that to fully account for the emergence of speech sounds in human infancy, consonant production must be addressed. Therefore, a number of models have now attempted to explain how combinations of vowels and consonants are acquired during early childhood [40, 41, 51–58]. The vast majority of these models assume from the very beginning of learning that speech production is already organized syllabically, and that the problem infants face is to fill in what vocal tract postures should occupy the consonant and vowel slots within that frame. It may be the case that once an infant has already reached the point of regularly producing canonical babbling this does reasonably approximate the type of learning they are performing. However, even if this is the case, such models do not address the question of how syllabic frames are themselves learned.

A noteworthy exception is a model by Moulin-Frier et al. [56]. This model does explicitly aim at an explanation of how syllabic babbling might emerge, positing that intrinsically motivated goal-setting could lead to a progression where infants first learn to phonate, then learn to produce vowels of different types, then learn to produce specific sequences containing both vowels and consonants. The model does move in the direction of more flexibility than a fixed consonant-vowel-consonant frame by programming movements using a set of five multidimensional Gaussian functions. However, Moulin-Frier et al.’s model operates at a higher level of abstraction than the model we present here. Moulin-Frier et al. do not attempt to relate the control of vocalization to neural dynamics.

Our model thus complements previous work on early vocal learning by addressing the question of how infants come to structure their vocalizations syllabically and by focusing on relating this to some key properties of human motor cortex; to do this we simplify the problem in a number of ways, leaving integration with processes explored in previous modeling work, such as imitation, intrinsically motivated goal-setting, perceptual-motor mapping, and multi-articulator control as a future direction [57].

Some initial studies using a spiking neural network and a setup very similar to that reported here showed a spiking neural network to be capable of learning to generate sounds that increased in syllabicity over the course of learning when reinforced by a human [59], and showed that reinforcement could be based on auditory salience [60]. The present study improves upon those initial studies in several ways, by modifying the neural architecture and making the reinforcement threshold increase as the model improves, both of which make the model’s learning more robust and better matched to what is observed in human development; increasing the number of simulations; exploring the influence of different parameter values; evaluating the model’s performance using an independent, automated metric of syllabicity; and exploring what types of activity patterns the model learns in order to increase its rate of canonical babbling production.

## Methods

**Fig 1:**
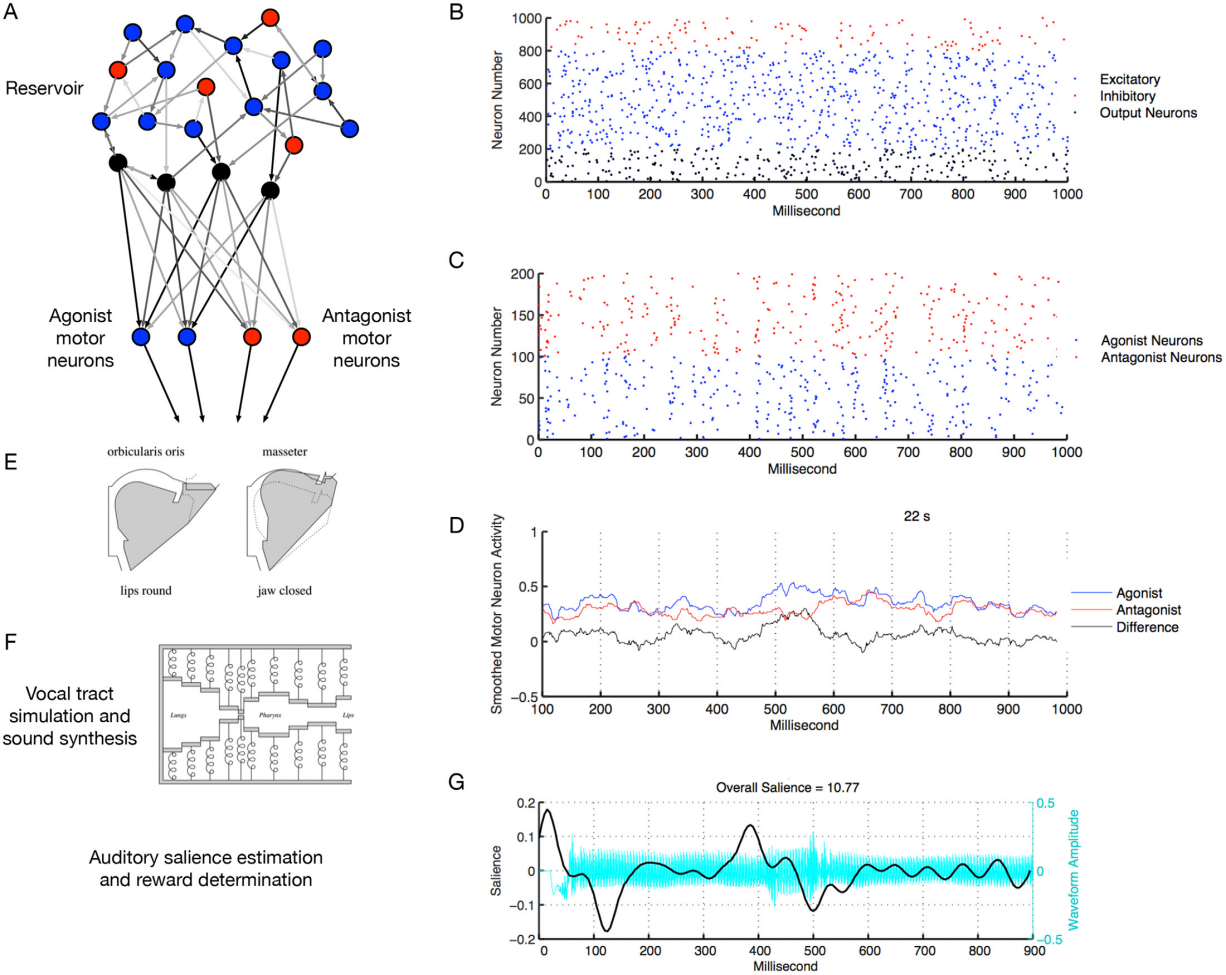
Overview of the model. A: Schematic depiction of the groups of neurons in the spiking neural network and how they are connected. There is a reservoir of 1000 recurrently connected neurons, with 200 of those being inhibitory (red) and the rest excitatory (blue and black). 200 of the reservoir’s excitatory neurons are designated as output neurons (black). These output neurons connect to two groups of motor neurons, agonist motor neurons (blue) and antagonist motor neurons (red). The connection weights within the reservoir are set at the start of the simulation to random values and do not change over the course of the simulation. The connection weights from the reservoir output neurons to the motor neurons are initially set to random values and are modified throughout the simulation by dopamine (DA)-modulated STDP. All reservoir and motor neurons receive random input current at each time step (not shown). B: Raster plot of spikes in the reservoir over a 1 s time period. C: Raster plot of spikes in the motor neuron groups over the same 1 s time period. The agonist and antagonist motor neuron spikes are summed at each time step then are smoothed using a 100 ms moving average. The smoothed antagonist activity is subtracted from the smoothed agonist activity, creating a net smoothed muscle activity that is sent to the orbicularis and masseter muscles. D: The smoothed agonist, antagonist, and net activity for the same 1 s as in the raster plots. E: Effects of the orbicularis oris and masseter on the vocal tract’s shape (reprinted with permission from [61]). Orbicularis oris activity tends to round and close the lips and masseter activity tends to raise the jaw. F: Schematic illustration that the vocal tract is modeled as an air-filled tube bounded by walls made up of coupled mass-spring systems (reprinted with permission from [61]). The orbicularis oris and masseter affect the equilibrium positions at the front parts of the tube. The air pressure over time and space in the tube is calculated, and the air pressure at the lip end of the tube forms the sound waveform. The vocal tract shape is modeled more realistically than depicted here and also contains a nasal cavity that is not depicted. G: The sound synthesized by the vocal tract model is input to an algorithm that estimates auditory salience. The plot shows, for the same 1 s as in B–D, the synthesized vocalization waveform (in cyan) and the salience of that waveform over time (in black). Apart from a peak in salience at the sound’s onset, the most salient portion of the sound is around the place where the sound’s one consonant can be heard. The overall salience of this particular sound is 10.77. If the salience of the sound is above the model’s current threshold, a reward is given, which causes an increase in dopamine concentration in the neural network.

Our model contains several components, illustrated schematically in Fig 1. The first component is a network of spiking neurons, itself divided into two subgroups. The neural network dynamically controls the muscle activities within a simulated vocal tract. The vocal tract simulation computes air pressures within the vocal tract, allowing sounds to be synthesized. The auditory salience of these sounds is then estimated, and auditory salience is used as the basis for whether or not the model receives a reward for producing a given sound. Reward engages Hebbian learning (via STDP) within the neural network. Each simulation was run for a total of 2 hours of simulation time, or 7200 trials each taking 1 s of simulated time. A number of simulations were run in order to choose appropriate model parameters and to assess the range of natural variation in performance across simulations. Each of these components is discussed in more detail below.

The simulation code is provided at https://github.com/AnneSWarlaumont/BabbleNN.

### Spiking neural network architecture

The neural network contained two main subgroups of neurons. The first subgroup was a reservoir of 1000 Izhikevich spiking neurons [62]. 80% of the neurons were excitatory and 20% were inhibitory. Each neuron was randomly assigned outgoing connections to 100 other neurons, with the constraint that inhibitory neurons could connect only to excitatory neurons. The reservoir neuron properties and synaptic connectivities were set almost identically to the network described in [63], and our simulation code incorporated MATLAB code from that work. See [64] for another example of an adaptation of such models to a reservoir architecture.

A subset of the excitatory neurons in the reservoir were selected to also connect to an equally sized subset of excitatory motor neurons, all having the same parameter values as the excitatory reservoir neurons. The motor neuron population had the same total number of neurons as the subset of the reservoir that projected to them. Half of the motor neurons were agonists, positively activating the masseter and orbicularis oris muscles and serving to promote closure of the jaw and mouth. The other half of the motor neurons were antagonists, inhibiting activity in the masseter and orbicularis oris muscles, thereby promoting jaw and mouth opening. Our assumption is that the reservoir and motor neurons can be considered as models of subgroups of neurons within motor regions of the neocortex. The motor neurons’ effects on the vocal tract muscles are intended to roughly model the influence of upper motor neurons on the muscles (via lower motor neurons).

The neural network simulation ran in millisecond simulated time increments. At each millisecond time increment, a random quantity of input current was given to each reservoir and motor neuron. Each neuron’s random input was drawn from a uniform distribution between -6.5 and 6.5 pA. This random input was the same as that given to the model in [63]; future work could test the implications of using other random input functions, such as exponential or power law input, and could aim to match this function to observations from real cortical neurons.

The random input current was added to the current that was given to each neuron due to the firings of the neuron’s presynaptic neurons that fired during the previous time step. The input current due to presynaptic neuron firing was proportional to a variable representing the synaptic strength from the presynaptic to the postsynaptic neuron. Some of these synaptic strengths (a.k.a. connection weights), the ones connecting the reservoir to the motor neurons, changed over the course of the simulation as a result of learning.

Note that there are no external inputs to the model other than the random inputs at each time step, which ensure spontaneous activity of the neurons in each group. This is by design, as the goal of the present work was to focus on how infants’ spontaneous vocalizations become more speech-like over the course of the first year of life (see [49] and [65] for further discussion).

### Vocalization synthesis

After every second of simulated time, a smoothed muscle activity time series was calculated. A 100 ms moving average of the previous 1000 ms time series of agonist motor neuron spikes was computed. The result was a 900 ms smoothed time series of agonist motor neuron activity. The same computation was done for the antagonist motor neuron spikes. The smoothed antagonist motor neuron activity time series was then subtracted from the smoothed agonist motor activity time series. The result was multiplied by a constant parameter, *m*, to create the net muscle activity time series. The scaling brought the muscle activity into a range that was appropriate for the synthesizer. The 900 ms net muscle activity time series was given directly to the articulatory vocalization synthesizer and specified both the Masseter and Orbicularis Oris muscle activities.

The vocalization synthesis relied on the articulatory synthesizer developed by Boersma and available in Praat [61, 66]. Praat version 5.3.32 for PC was used for all the simulations. The synthesizer models the walls of the vocal tract as a set of coupled, damped mass-spring systems whose equilibrium positions and spring constants are affected by the activation of the various vocal tract muscles. The air within the vocal tract is treated as a fluid whose aerodynamics are modeled by obtaining approximate numerical solutions to a set of equations representing constraints such as conservation of mass, response to pressure gradients, and friction. The air within the vocal tract affects the movements of the walls and vice versa.

Besides the Masseter and Orbicularis Oris activity, a number of other parameters needed to be set in order to generate the synthesized vocalizations. The speaker type needed to be specified; we chose the adult female vocal tract model for all simulations. Although Praat does have a child vocal tract model, it does not have a built-in infant model. Additionally, for the child model to generate sound, the acoustic simulation sampling rate must be increased. This would increase the computational demands of the vocalization synthesis, which is already the main processing bottleneck within our model. Since the focus of this study was on neuromotor learning rather than on infant vs. adult anatomy, we reasoned that the adult female vocal tract provided a reasonable enough approximation of the main bioacoustic constraints on the infant vocal tract, particularly the nonlinear relationships of jaw and mouth movement to vocalization acoustics, for our purposes. The default sampling rate, 22050 Hz, was used. For each sound, the Lungs parameter, which specifies the target lung volume, was set to 0.1 at 0 ms, to 0.1 at 20 ms, to 0 at 50 ms, and to 0 at 900 ms. This created a scenario where the target lung volume went quickly from a high value at the beginning of the vocalization to a low value a few tens of ms later. In a human, such a change would be due to coordinated activity of the muscles of the diaphragm and rib cage. One laryngeal muscle, the Interarytenoid, was set to a value of 0.5 for the duration of the 900 ms vocalization. This muscle has the effect of adducting the vocal folds, causing a pressure differential between the lungs and the upper vocal tract that sets the vocal folds into vibratory motion. Finally, the Hyoglossus muscle, which lowers the tongue, was set to a value of 0.4 throughout the 900 ms vocalization. This made the vocal tract such that when the jaw and lips were open, the vocalization would sound like the vowel [A]. (See [49] for an example of a model that learns the settings of the laryngeal muscles for static, vowel-only vocalizations.)

This combination of 900 ms of muscle activations and other settings was sent to the vocal tract model, which simulates the air pressure throughout the vocal tract at a series of time points and uses the time series of pressures at the mouth of the vocal tract to synthesize the vocalization. The vocalization was saved as a WAV file and subsequently analyzed to estimate its auditory salience.

### Auditory salience and reward

The estimated auditory salience of each sound was used as the basis for determining when to reward the model. This was based on the idea that human infants will tend to prefer more salient stimuli as well as on the idea that human caregivers are more likely to notice and respond to more salient infant sounds.

Salience was estimated using a program developed by Coath, Denham, and colleagues [36, 37]. The program takes a sound as input and analyzes that sound in a variety of ways. It first converts the sound to a spectrogram format, with the frequency and time bins based on a model of cochlear processing. Within that cortical response spectrogram, it then identifies points in time and frequency where there are transitions in the cochlear activity level. This is essentially a form of temporal edge detection. After that, it convolves the spectrotemporal transients with models of cortical filters. The cortical filter models were developed by unsupervised training on a corpus of speech data. The cortical filters are designed to well represent the input data with minimal redundancy. The final step in the salience estimation was to detect transients in the activation of these cortical filter models. Both onset transients and offset transients are detected. The transients can be thought of as auditory edge detectors [37]. The overall amount of change in the cortical filter activations at a series of evenly spaced time points determined the salience function for the particular input sound.

The salience, *s*(*v*, *t*), over time, *t*, for a given second’s vocalization, *v*, was then converted to a single overall salience score for the sound, *S*(*v*), by taking the sum of the absolute value of the salience function the sound (so as to include both onset and offset transients), excluding the first 150 ms:

$$S\left(v\right)=\sum_{t=151\text{ms}}^{900\text{ms}}\left|s\left(v,t\right)\right|$$ (1)

The first 150 ms were excluded because they typically included a spike in salience related to the abrupt onset of the sound, and this spike was not related to the questions of interest in the present study.

The model received a reward if the salience for the sound it had just produced, *S*(*v*), was greater than a threshold value, *θ*(*v*). The threshold was initialized to a value of 4.5 and increased as the model increased the salience of its productions. If on the last 10 trials at least 30% of the model’s vocalizations were rewarded, the threshold value was increased by 0.1. (See Algorithm 1.) The starting threshold, threshold increment, and 30% criteria were decided based on informal explorations during development of pilot versions of the model.

**Algorithm 1:** Adapting the reward threshold.

1:	*θ* ← 4.5	▷ Initialize the reward threshold.
2:	*h*[1: 10] ← [0, 0, 0, 0, 0, 0, 0, 0, 0, 0]	▷ Initialize the recent reward history.
3:	**for** each second’s vocalization **do**	
4:	**if** *S* > *θ* **then**	▷ If salience is high, reward.
5:	*r* ← 1	
6:	**else**	
7:	*r* ← 0	
8:	*h*[11] ← *r*	▷ Update the recent reward history.
9:	*h* ← *h*[2: 11]	
10:	**if** $\sum_{n=1}^{10}h\left[n\right]\geq 3$ **then**	▷ If the recent reward rate is 30% or higher
11:	*θ* ← *θ* + .1	▷ increase the reward threshold
12:	*h*[1: 10] ← [0, 0, 0, 0, 0, 0, 0, 0, 0, 0]	▷ and reset the recent reward history.

### Neural connections and learning

At the beginning of the simulation all neural connection weights within the reservoir were assigned random values. The outgoing connection weights from the excitatory neurons were drawn from a uniform random distribution between 0 and 1. The outgoing connection weights from the inhibitory neurons were drawn from a uniform random distribution between -1 and 0. These connection weights remained the same throughout the simulation. All initial connection weights between the reservoir and the motor neurons were drawn from a uniform random distribution between 0 and 1.

The connections from the reservoir neurons to the motor neurons were updated via reward-modulated spike-timing-dependent plasticity. Spike-timing-dependent plasticity (STDP) is a form of Hebbian learning derived from a large number of both in vitro and in vivo studies on long term potentiation and depression, in both hippocampal and neocortical neurons [67, 68]. In STDP, the change in strength of a synapse connecting a presynaptic neuron to a postsynaptic neuron is related to the relative timing of spikes of those two neurons. Long term potentiation occurs when the presynaptic neuron fires before the postsynaptic neuron and long term depression occurs when the presynaptic neuron fires after the postsynaptic neuron. The degree of potentiation or depression is greater the closer together the two spikes are. There is evidence that the presence of dopamine increases learning rates in the neocortex and that such dopamine-modulated long term potentiation in the motor cortex facilitates skill acquisition [69–71]. It is believed that this provides a means by which animals learn to recreate movement patterns that lead to rewarding outcomes.

Izhikevich’s DA-modulated STDP algorithm [63] was used, with the modification that in our model only the long term potentiation aspect of STDP is implemented. Rather than implement spike-timing dependent long term depression, the reservoir to motor neuron connection weights are periodically normalized. The algorithm is presented in Algorithm 2 and its essential features are described in the following paragraph.

**Algorithm 2:** Reward-modulated spike-timing-dependent plasticity.

1:	*d* = 0	▷ Dopamine concentration
2:	**for all** reservoir output neurons, *r*, **do**	
3:	*c*_*r*_ = 0	▷ Trace of *r*’s previous firings
4:	**for all** motor neurons, *m*, **do**	
5:	*e*_*rm*_ = 0	▷ Eligibility trace
6:	draw *s*_*rm*_ from *U*(0, 1)	▷ Strength of synapse of *r* onto *m*
7:	**for all** milliseconds of simulation time, *t*, **do**	
8:	*d* = .995 * *d*	▷ Dopamine concentration decays exponentially
9:	**for all** *r* **do**	
10:	**for all** *m* **do**	
11:	**if** *m* spikes **then**	
12:	*e*_*rm*_ = *e*_*rm*_ + *c*_*r*_	▷ Eligibility trace increases
13:	**if** the remainder of *t*/10 is zero **then**	▷ Every 10 ms
14:	*s*_*rm*_ = *min*(*s*_*rm*_ + *e*_*rm*_ * *d*, 4)	▷ Synapse strength increases
15:	**if** *r* spikes **then**	
16:	*c*_*r*_ = .1	▷ Set memory of *r* spiking to its max value
17:	*c*_*r*_ = .95 * *c*_*r*_	▷ Memory of *r* spiking decreases exponentially
18:	**if** the remainder of *t*/10 is zero **then**	
19:	*S* = ∑_*r*_∑_*m*_ *s*_*rm*_	
20:	**for all** *r* **do**	
21:	**for all** *m* **do**	
22:	*s*_*rm*_ = *s*_*rm*_/*S*	▷ Normalize the synaptic strengths
23:	*e*_*rm*_ = .99 * *e*_*rm*_	▷ Eligibility trace decays exponentially
24:	**if** reinforced for producing a high-salience sound **then**	
25:	*d* = *d* + 1	▷ Dopamine increases

Each time an output neuron within the reservoir spikes, a small amount, 0.1, is assigned to a trace memory of the firing of that neuron. These reservoir output neuron traces decrease exponentially with time. Whenever a motor neuron fires, the eligibility trace for each of its incoming synapses to be strengthened is increased by adding the memory traces of the firings of the reservoir output neurons. This eligibility trace is then multiplied by the dopamine level in order to determine how much the synapse strength is increased. The dopamine level is increased by adding 1 whenever a reward is received. The dopamine level, eligibility traces, and presynaptic firing memories decay exponentially over time. At each synaptic weight update, if the update would make the strength of the synapse greater than 4, the synaptic strength is capped at 4. This prevents any individual synapse from becoming overly, and unrealistically, strong. Due to the nature of the learning algorithm, no synapse strength could ever have a negative value. Finally, after each synaptic weight update, the synaptic weights are normalized by dividing all weights by the mean synapse strength. This prevents the overall network connectivity from increasing over time, which would severely disrupt the network’s dynamics [72]. Based on pilot explorations, this method of normalization seemed to be less sensitive to small parameter variations than relying solely on long term depression to keep synapse strengths within a desirable range; further exploration of this issue is warranted but outside the scope of the present study. Note that the reward function and the DA-modulated STDP were both deterministic. All random variation in the model stemmed from the random synaptic weight initialization and the random input currents given to the neurons.

### Simulation sets

Pilot explorations indicated that the types of sounds that are generated by the model are particularly sensitive to two parameters, the number of motor neurons and the muscle activity scaling parameter, *m*. With larger numbers of motor neurons in both the agonist and antagonist groups, the net motor neuron activity level tends to exhibit higher amplitude variation within a second, i.e. within a vocalization. This leads to a greater likelihood of syllabic vocalizations, since the jaw and lip muscle activities tend to vary within a greater range. For the same reasons, when the muscle scaling parameter, *m*, which is multiplied by the net motor neuron activity to generate muscle activity, is higher, the range of jaw and lip muscle activities tends to vary more greatly within a vocalization, leading to more syllabic vocalizations.

To demonstrate this, and to determine appropriate values of these two parameters for focusing more detailed analyses, we ran 13 sets of simulations, varying the number of motor neurons and the value of the muscle scaling parameter, *m*. Each set of simulations consisted of 5 simulations with different random synaptic weight initializations and different random inputs given at each time step to the reservoir and motor neurons. We explored three values of the number of motor neurons: 50, 100, and 200. The number of reservoir output neurons was matched to the number of motor neurons, so that as the number of motor neurons increased, the output neurons in the reservoir also increased. The number of agonist motor neurons and the number of antagonist motor neurons were always equal, so if the total number of motor neurons was 50, this meant there were 25 agonist motor neurons promoting jaw and lip closure and 25 antagonist motor neurons promoting jaw and lip opening. We initially explored three values of *m*: 4, 5, and 6. Recall that *m* is the value that the difference between the smoothed agonist motor neuron spike counts and the smoothed antagonist motor neuron spike counts is multiplied by in order to obtain the time series of masseter and orbicularis oris muscle activities. We tested every pairwise combination of these number of motor neurons and values of *m*, making for 9 different parameter combinations in total. Based on the results of these 9 simulation sets, we then decided to test four additional parameter combinations, to cover 50 neurons with *m* = 7 and 8 and 200 motor neurons with *m* = 2 and 3. This made for a total of 13 parameter combinations tested. We then took the combination that appeared to provide the best combination of learning capability and realism in the initial behavioral starting point (200 neurons and *m* = 2), and focused further analyses on the simulations with that parameter combination.

### Yoked controls

Even with the synaptic weight normalization in place, changes in the neural network’s connection weights due to STDP can potentially lead to changes in network dynamics that could affect the oscillatory dynamics of the motor neuron population and in turn the types of vocalizations the model produces. In addition, even with no synaptic weight changes, over time there can be subtle changes in the neural dynamics.

To ensure that salience-based rewards are driving any increases in vocalization salience and canonical syllable production over time, we ran yoked control simulations. These were simulations with their own unique random synaptic weight initializations and random inputs at each timestep, but with reward times taken from a previous simulation in which rewards were salience-driven. This matched the timings of synaptic modification to those of the real simulations, while making yoked control model rewards uncorrelated with the salience of vocalization. This control method is standard procedure in work on animal behavior, including experimental work on human vocal learning during the first year of life (e.g., see [73]).

### Syllable estimation

In previous work [59, 60], the syllabicity of the sounds produced by a similar model had been evaluated using two metrics. The first was the salience of the sounds. Based on previous work showing human ratings of the syllabic quality of a sound to be correlated with our auditory salience metric, as well as theoretical considerations of the concept of auditory salience and the specific auditory salience estimation algorithm used here, we expected this to be a fairly useful metric. We also listened ourselves to the sounds produced by the model, to verify with our own ears what the vocalizations sounded like and how they compared to infants’ syllabic and non-syllabic vocalizations (links to sound examples that the reader can download are given in the Results section, and examples of human infant vocalizations classified as canonical, i.e. syllabic, vs. non-canonical are available at www.babyvoc.org through the IVICT tool).

To provide an additional metric of the syllabicity of the sounds, as well as a metric that was independent of the development of the computational model, we utilized a Praat script for automatically identifying syllable nuclei in adult speech, developed by de Jong and Wempe [74, 75]. This syllable detection algorithm uses a combination of amplitude difference and voicing information to estimate where syllable nuclei, i.e the loudest parts of a syllable, usually the part containing the vowel, occur. It first searches the sound for segments where there is a high amplitude portion surrounded by lower amplitude sound. It then checks that there is an identifiable pitch, i.e. the perceptual correlate of fundamental frequency, during the high amplitude portion. If so, it labels this a likely syllable nucleus. We ran this program using the model’s individual 900 ms vocalizations as input, and, for each input vocalization, obtained the total number of syllable nuclei that the sound was estimated to contain. We used all the default parameters, i.e. a silence threshold of -25 dB, a minimum dip between peaks of 2 dB, and a minimum pause duration of 0.3 s.

## Results

### Examples of model vocalizations

**Fig 2:**
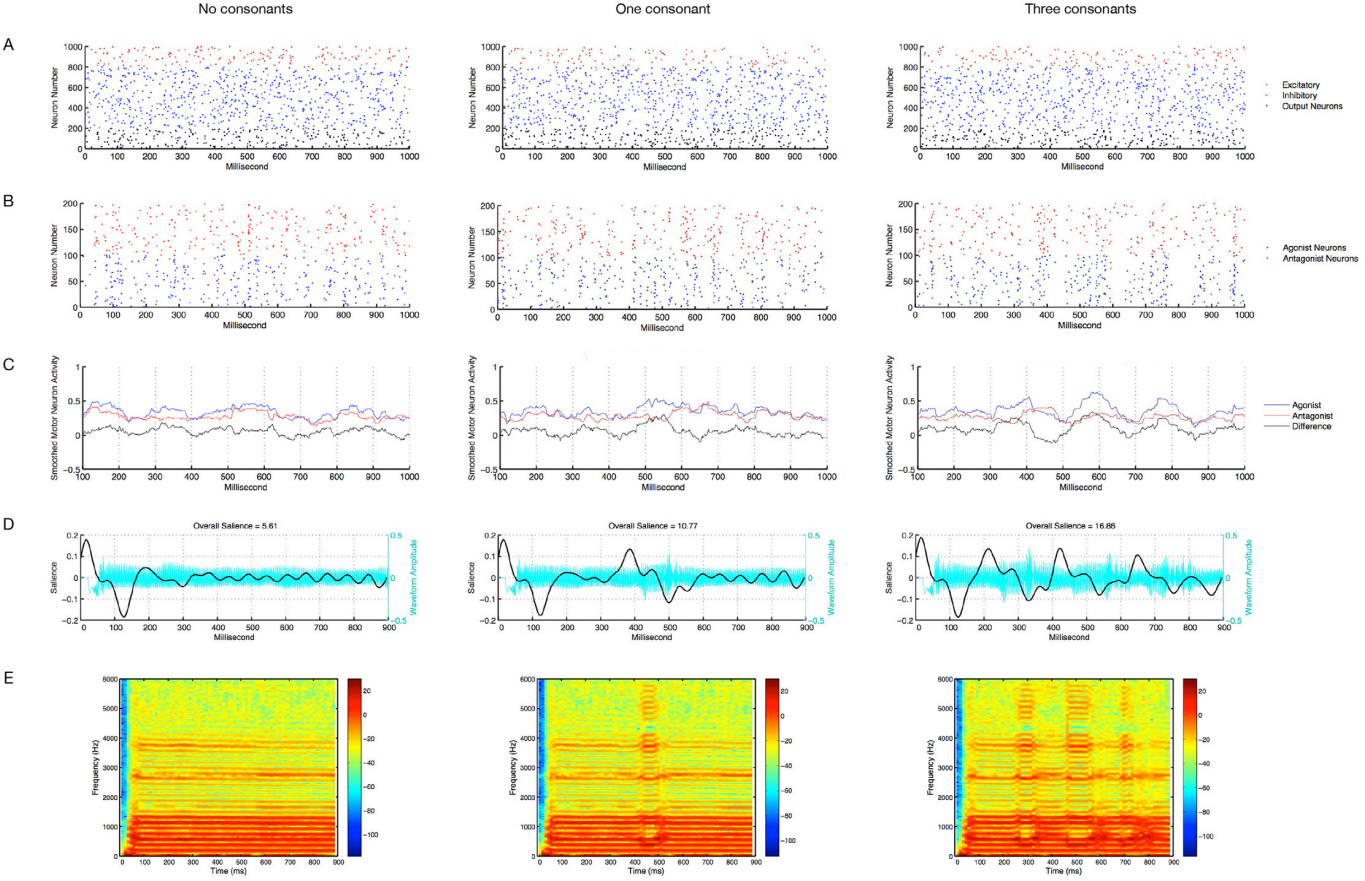
Vocalization examples. Three examples of vocalizations produced by the model. The left column shows a vocalization that contains no consonants and would not be considered canonical or syllabic babbling. The associated WAV file is available for listening in S1 Sound. The middle column shows a vocalization that contains one consonant and the right column shows a vocalization that contains three consonants. The middle and right vocalizations would qualify as canonical babbling (the associated WAV files are available for listening in S2 Sound and S3 Sound, respectively). The vocalizations were all produced by fully trained versions of the primary version of the model. A: Raster plots of the 1 s of reservoir neuron activity associated with the vocalization. B: motor neuron raster plots. C: Smoothed motor neuron activity for the agonist and antagonist groups as well as the difference between the smoothed agonist and antagonist activities. This difference was what was input as muscle activity to the vocalizations synthesizer. D: Waveforms (cyan), salience traces (black) and overall salience estimates (titles) for each example vocalization. Note that positive values of the salience trace represent detection of onsets of patterns in the auditory stimulus and negative values represent offsets of patterns. E: Spectrograms of the vocalizations; these provide visual evidence of the vocalization’s harmonic frequencies and of formant transitions associated with the production of consonants.

Fig 2 and S1 Sound, S2 Sound and S3 Sound provide three examples of vocalizations produced by the model, one non-syllabic (the most primitive), one syllabic with a single consonant, and one syllabic with multiple consonants (the most advanced). The consonants are apparent as amplitude fluctuations in the sound waveforms and as amplitude and formant shifts in the spectrograms. The plots of the salience of the sounds over time show how consonant productions are often associated with peaks in the estimated salience. The more salience peaks there are, and the more dramatic they are, the larger the overall salience of the sound tends to be. The net motor activity that serves as input to the muscles tends to show peaks around the time of the consonant productions, reflecting the increased activity of the orbicularis oris and masseter muscles that bring the mouth to a more closed position to create a consonant sound.

### Dependence of results on number of motor neurons and muscle scaling

Table 1 shows the average salience of sounds produced at the start and end of the simulation for each combination of motor neuron number and muscle scaling, *m*. The start of learning was defined as the first 60 vocalizations produced by the model, which corresponded to the first minute of simulation time. End of the simulation was defined as the last 60 vocalizations produced by the model, which corresponded to the last minute (minute 120) of simulation time.

**Table 1:** Results using different parameter combinations.

	50 neurons	100 neurons	200 neurons
scale by 2			start: 5.0 (0.24) end: 9.7 (0.53)***
scale by 3			start: 7.0 (0.48) end: 11.3 (0.32)***
scale by 4	start: 4.5 (0.07) end: 4.5 (0.04)	start: 5.9 (0.40) end: 8.2 (0.47)***	start: 9.5 (0.60) end: 12.5 (0.55)**
scale by 5	start: 4.8 (0.12) end: 5.1 (0.16)	start: 7.0 (0.56) end: 10.0 (0.58)***	start: 11.5 (0.27) end: 12.8 (0.28)***
scale by 6	start: 5.4 (0.19) end: 6.3 (0.29)**	start: 8.6 (0.54) end: 10.9 (0.26)**	start: 12.7 (0.48) end: 12.5 (0.75)
scale by 7	start: 6.4 (0.56) end: 7.2 (0.22)*		
scale by 8	start: 7.4 (0.33) end: 8.3 (0.62)*		

Beginning and ending salience as a function of number of output and motor neurons and scaling from motor activity to muscle activity. The start values are the average salience over the first 60 sounds (i.e. the first simulated minute’s worth of vocalization) for the five simulations using that cell’s parameter combination. The end values are the average salience over the last 60 sounds (i.e. the 120th simulated minute’s). Standard deviations of the mean values across simulations are in parentheses. Asterisks indicate where a paired t-test found a significant difference between the average Minute 1 salience and the average Minute 120 salience across the five simulations, * *p* < .05, ** *p* < .01, *** *p* < .001. Blank cells indicate parameter combinations that were not tested.

The two parameters we manipulated were related to variations in both starting and ending salience as well as being related to how much learning, i.e. increase in salience, the model exhibited. For the 50 motor neuron simulations, the model only exhibited learning when the muscle scaling parameter, *m*, had higher values, 6, 7, or 8, and even then the increase in salience was small. When 100 motor neurons were used, the model exhibited learning with moderate salience increases for all three values of *m*. For the 200 motor neuron simulations, the model exhibited large salience increases when *m* was small (2, 3, and 4), demonstrated learning with moderate salience increases when *m* had a value of 5, and did not show any learning when *m* had its highest value, 6.

The value of *m* appears to primarily affect whether the starting and ending salience tended to be on the lower or higher side, with lower values of *m* associated with lower salience and higher values of *m* associated with higher salience. The number of output and motor neurons also affects whether salience tends to be overall lower or higher. These effects on starting salience could be quite extreme. For example, in the case of scaling by 6 with 200 output and motor neurons, the starting salience was 12.7, which is higher than or as high as the ending salience for any of the other parameter combinations. As will be made clearer below, when the relationship between motor variability and salience is explored, the high starting salience for the high *m* and high neuron number simulations can be expected given that both parameters will increase the amplitude of muscle activity oscillation. Very high amplitude oscillations will lead to frequent oscillations between mouth opening and closure, yielding highly syllabic sounds. The question of why infants do not simply begin life generating high degrees of lip and jaw oscillation is addressed in the Theoretical Implications section of the Discussion.

Both variables also appear to affect the general amount of learning, defined here as quantity of change in salience. It appears to be the case that when *m* is too low or too high for a given neuron number, the model’s performance is at floor or ceiling, respectively. The number of neurons appears to have a more graded effect on the degree of learning that takes place. Provided *m* is within an acceptable range, larger neuron numbers tend to increase the degree of leaning.

The parameter exploration revealed a number of potential candidate parameter combinations for use in further exploration and analysis, in that several of the parameter combinations did exhibit substantial learning, in particular the 200 motor neuron simulations with *m* = 2, 3, or 4. However, exhibiting some learning and substantial increase in salience over time is not the only relevant factor. It is also important that the model start from a realistic starting point, i.e., one in which the model does not exhibit much if any canonical babbling. This matches the fact that prior to about 6 months, most infants almost never produce canonical syllables, except rarely and apparently accidentally. In other words, like human infants, a model of canonical babbling development should start from a state of not regularly producing canonical syllables, and from there should acquire the ability to produce canonical syllables progressively more frequently.

To aid in choosing a parameter combination to explore further, the vocalizations produced by each simulation from each parameter combination were sampled at 5 minute intervals. For each simulation, these samples were concatenated into a single sound file that provides an auditory sense of how the simulation’s vocalizations changed over time. Based on listening to the sounds produced by the different parameter combinations at the beginning and the end of the simulation period, in terms of meeting both criteria, i.e., having a realistic starting point and showing increased canonical babbling over time, the best parameter combination was 200 motor neurons and *m* = 2. The other combinations that showed high increase in salience (200 motor neurons and *m* = 3 or 4) started from a point of already producing canonical syllables fairly frequently. Thus, in the rest of the Results section, we will focus on the 200 output/motor neuron and *m* = 2 simulations.

The five sound files that give snapshots of this best parameter combination’s vocalizations over the course of learning are available in S4 Sound, S5 Sound, S6 Sound, S7 Sound and S8 Sound. Snapshots for the corresponding yoked control simulations are available in S9 Sound, S10 Sound, S11 Sound, S12 Sound and S13 Sound. The full set of sound files for all parameter combinations is available at http://dx.doi.org/10.6084/m9.figshare.1486454.

### Evidence for the model’s learning

This section describes the results of statistical tests of whether the model learned over time compared to the yoked control simulations. We operationalized learning as an increase in salience of the sounds and as an increase in average number of syllables. Recall that salience was also the basis for model reinforcement whereas the automatic syllable detector was not applied to the model vocalizations until completion of the simulations. Thus, the salience metric shows how the model performed relative to its training criterion and number of syllables provides a more independent measure of whether the model actually increases the number of syllables it produces.

**Fig 3:**
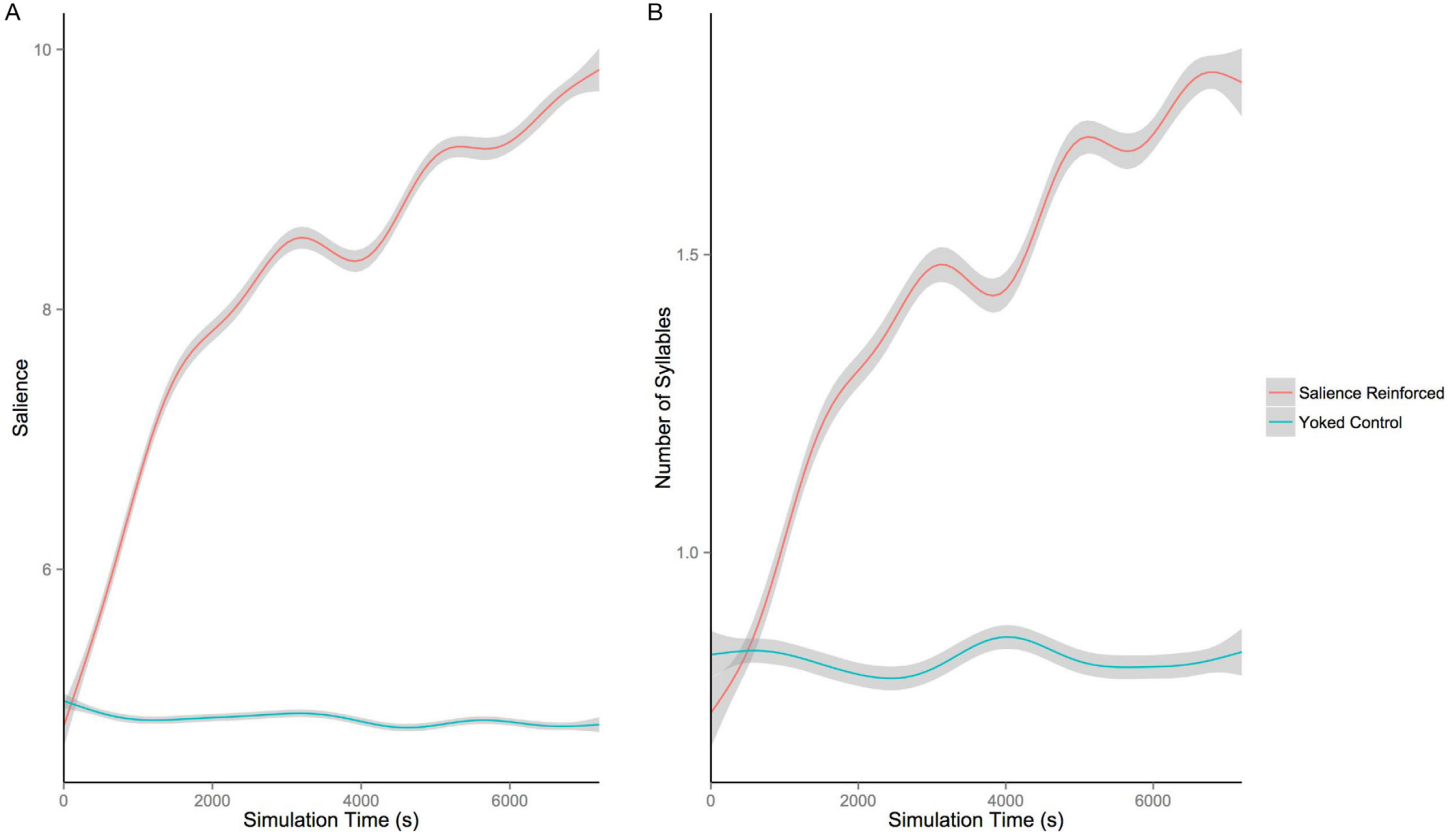
Increase in salience and syllabicity over time. A: Average auditory salience of the sounds produced by the model as a function of simulation time in seconds and whether the simulation was reinforced based on auditory salience or was a yoked control. B: Number of vowel nuclei, i.e. number of syllables, estimated to be contained within the sounds produced by the model as a function of simulation time in seconds and whether the simulation was reinforced based on auditory salience or was a yoked control. Lines are generalized additive model fits and dark gray shading gives 95% confidence intervals around those fits. When reinforced for auditory salience, the model increases both the salience of its vocalizations and the number of syllables contained within those vocalizations, while the yoked controls do not show such increases.

Fig 3A shows the salience of the vocalizations produced by the model as a function of simulation time and in comparison to yoked control simulations. A linear mixed effects model predicting a vocalization’s salience with simulation number as a random effect and simulation time, yoked control status, and the interaction between simulation time and yoked control status as fixed effects indicated that there was an increase in vocalization salience over simulation time, *β* = 0.42, *p* < . 001 and that salience was greater for vocalizations produced by salience-reinforced model simulations compared to those produced by their yoked control simulations, *β* = 1.2, *p* < .001. There was also a statistically significant interaction between whether the simulation was a yoked control simulation and simulation time, *β* = 0.43, *p* < .001, reflecting the fact that the salience-reinforced version of the model increased its vocalization salience over time whereas the yoked control version of the model did not.

As can be seen in Fig 3B, the number of syllables produced by the salience-reinforced model increased with simulation time whereas the syllabicity of the yoked control simulations’ productions remained fairly constant over time. A linear mixed effects model predicting number of syllables with simulation number as a random effect and time, yoked control status, and the interaction between time and yoked control status as fixed effects indicated several statistically significant patterns. There was an increase in number of syllables over time, *β* = 0.31, *p* < .001, a higher number of syllables for salience-reinforced simulations compared to yoked controls, *β* = 0.66, *p* < .001, and an interaction such that salience-reinforced simulations exhibited more increase in syllabicity over time than yoked control simulations, *β* = .31, *p* < .001. Compared to the yoked control simulations, the salience-reinforced simulations produced about two syllables per vocalization after the two hours of simulation time, compared to a baseline of less than one syllable per vocalization.

### How does the neural network increase vocalization salience?

Having established that the model’s learning increases both the salience and the average number of syllables of its vocalizations, the next question is how the model learns to do this. We analyzed the changes that took place in the model’s muscle activity time series to see what features of its motor neuron dynamics changed during learning. We also visualized the changes in synaptic connection weights at the beginning compared to the end of simulation.

We characterized the 900 ms muscle activity time series associated with each vocalization in terms of two features. The first was the standard deviation of the 900 values in the time series. The standard deviation of the muscle activity would be expected to correlate with the quantity of movement of the jaw and lips. Amount of movement is expected to relate positively with the salience of a sound, as more movement should generate greater sound change.

The second feature was the mean activity level over the 900 ms. This will tend to correlate with the base position of the jaw and lips around which any movement takes place and will also have an effect on the sounds the vocal tract model produces. When the mouth is slightly open, increase in masseter and orbicularis oris activity can easily cause the mouth to close, stopping the air flow and creating a consonant sound, and decrease in masseter and orbicularis oris activity will tend to cause the mouth to move toward facilitating production of a more open vowel sound. Thus, mean masseter and orbicularis oris activity that places the mouth in a slightly open position is likely the ideal scenario for generating canonical babbling sounds. If the mean activity of these muscles is too great, the mouth will be constantly in a closed position and changes in muscle activation around this mean level will not change the fact that the mouth is closed; this will tend to lead to sounds with low salience scores that do not contain consonant-vowel alternations. At the other extreme, if the baseline masseter and orbicularis oris activity are very low, this will tend to position the mouth rather wide open, and it will take much greater movement for the mouth to close enough to generate a clear consonant sound; the typical range of movement may tend to lead to slight changes in vowel type instead.

**Fig 4:**
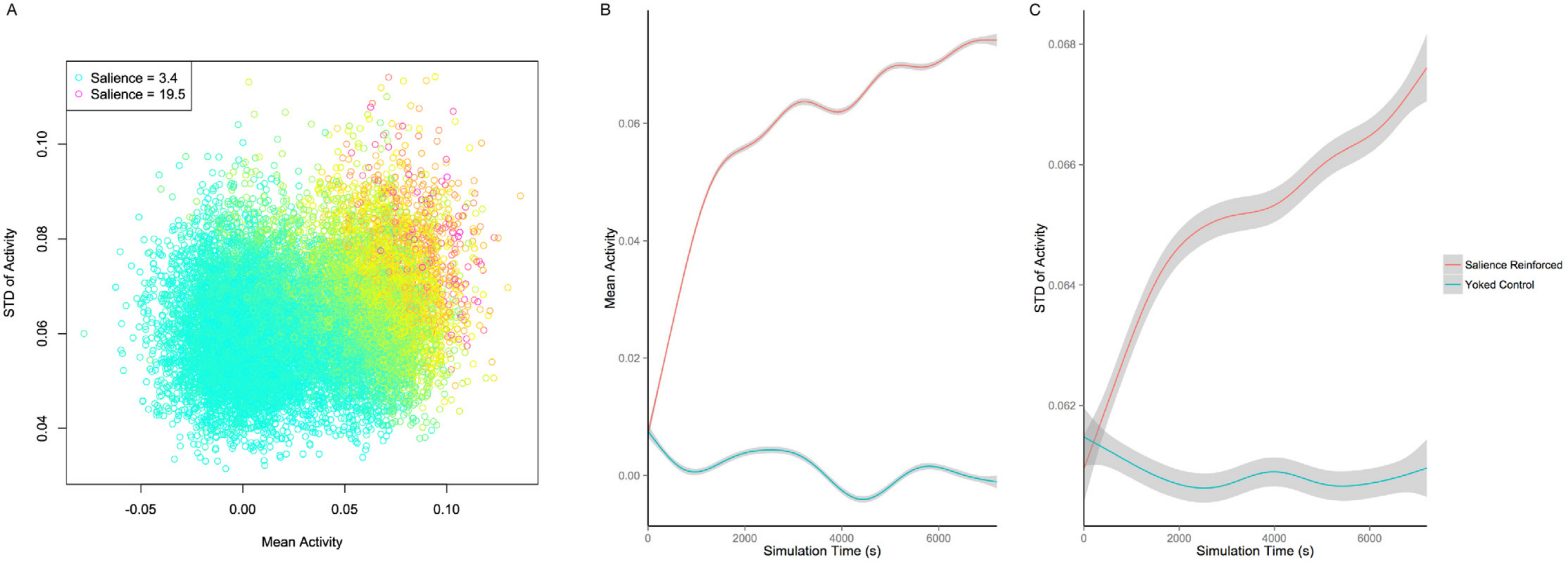
The relationship of muscle activity mean and standard deviation to salience and learning. A: Each point represents one vocalization produced by five simulations of the salience-reinforced model. Data are sampled so that every fifth vocalization produced by the model is plotted here. Note that the most salient sounds tend to have both high median activity levels and high standard deviation of muscle activity, as our statistical analyses indicate. The legend shows the colors of the maximum and minimum salience points portrayed in the plot; red indicates high salience, yellow indicates moderate salience, and cyan indicates low salience. B: The mean level of muscle activity produced by the model as a function of simulation time in seconds and whether the simulation was reinforced based on auditory salience or was a yoked control. Lines are generalized additive model fits and dark gray shading gives 95% confidence intervals around those fits. When reinforced for auditory salience, the model increases the baseline level of activity of the masseter and orbicularis oris muscles, leading to greater mouth closure on average after learning. The yoked controls do not show such an increase. C: The average, across vocalizations, of the standard deviation of muscle activity within each vocalization, as a function of simulation time in seconds and whether the simulation was reinforced based on auditory salience or was a yoked control. The salience-reinforced model increases its within-vocalization change in activity of the masseter and orbicularis oris muscles, leading to greater jaw and lip movement on average after learning.

To test these ideas for how standard deviation and mean of the activity time series should relate to the sounds the vocal tract model produces, we ran a multiple regression with standard deviation and mean as predictors and salience as the dependent variable to be predicted, with all sounds from all five simulations and their yoked controls as data points. As expected, standard deviation was positively associated with salience, *β* = 0.367, *p* < .001. Mean activity level was also positively associated with salience, *β* = 0.671, *p* < .001. The relationship of salience to muscle activity standard deviation and mean is depicted graphically in Fig 4A. This suggests that most of the vocalizations were on the side of having lower than desirable baseline and range orbicularis oris and masseter activity, so that increasing the baseline activity level of these muscles, which would have to be accomplished through greater agonist motor neuron activity, would lead to increasing vocalization salience and increasing likelihood of generating consonant sounds.

Given these associations between standard deviation of activity and salience and mean activity level and salience, did the neural network model effectively learn to increase either or both of these features in order to increase the salience of its vocalizations? A mixed effects model predicting standard deviation of muscle activity with simulation number as a random effect and simulation type (real vs. yoked control), simulation time, and interaction between simulation type and simulation time as fixed effects revealed that the real simulation had greater standard deviations than the yoked control, *β* = 0.371, *p* < .001, that as simulation time increased, the standard deviation increased, *β* = 0.129, *p* < .001, and that there was an interaction between the two factors, *β* = 0.138, *p* < .001. Based on a regression with the same predictor variables but mean activity level as the dependent variable to be predicted, the real simulations had significantly higher mean activity levels than the yoked control simulations, *β* = .161, *p* < .001, there was a significant positive effect of simulation time, *β* = 0.378, *p* < .001, and there was a significant interaction between the two factors, *β* = 0.419, *p* < .001. These results can be visualized in Fig 4B and 4C. These results indicate that the model did increase both the amount of muscle activity variation and the baseline level of muscle activity over the course of learning. The change in baseline activity is consistent with the phonetic research demonstrating that there are greater changes in vocal tract resonances when a change in vocal tract aperture occurs while the vocal tract is nearly closed than when the same change in aperture occurs while the vocal tract is relatively open [76].

**Fig 5:**
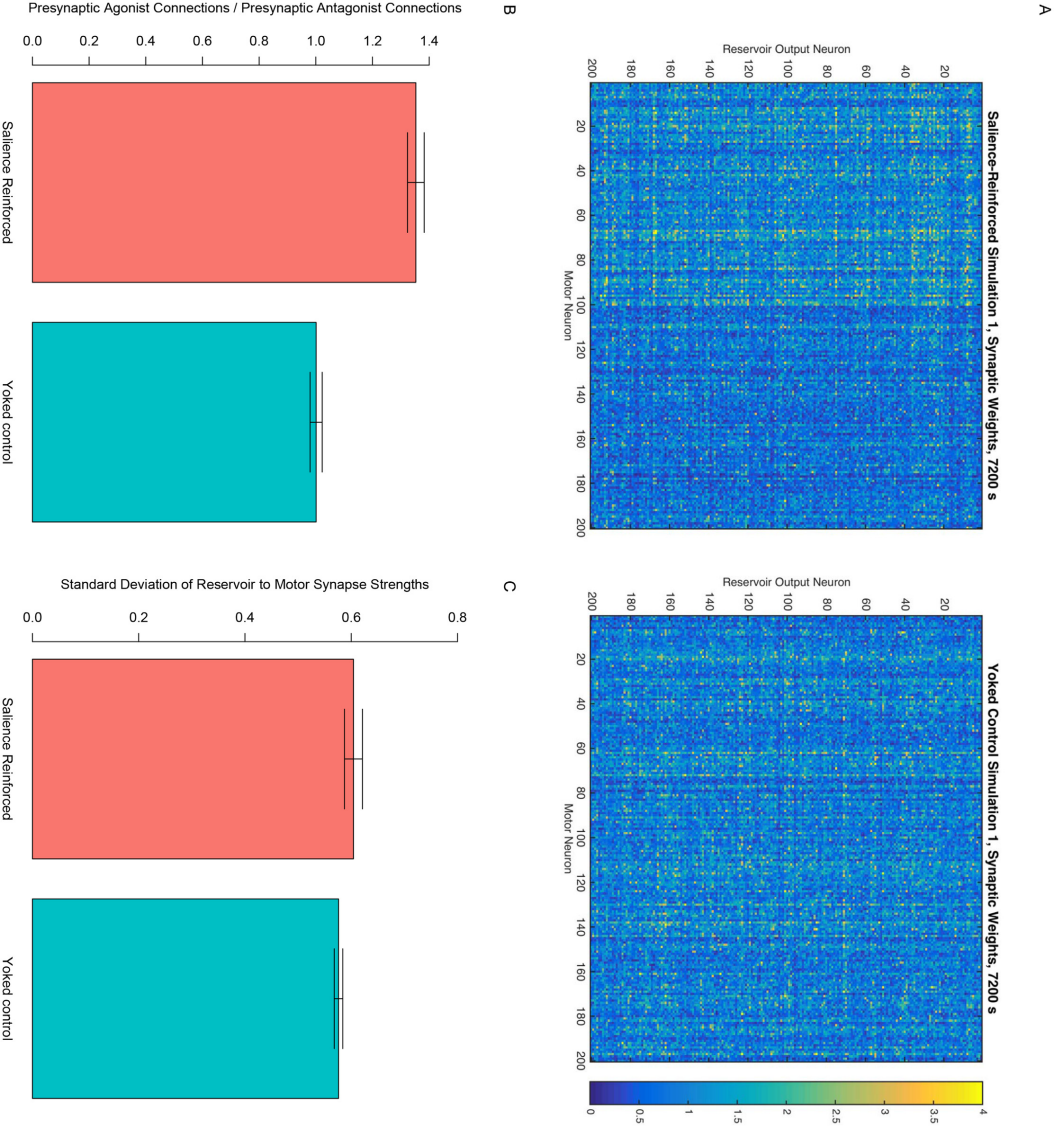
Synaptic weights after learning. A: Example of the synapse strengths from each reservoir output neuron to each motor neuron after learning. The left plot shows the synapses for the first simulation of the 200 motor neuron *m* = 2 model reinforced for high-salience vocalizations. The right plot shows the synapses for the corresponding yoked control simulation. Yellow indicates greater connection strengths; blue indicates weaker synapses. The stronger synapses on the left half of the left plot as compared to the right half of that same plot reflect the greater connection of reservoir neurons to agonist motor neurons promoting mouth closure than to antagonist motor neurons promoting mouth opening. Note that this bias is not present in the connection weights of the yoked control simulation shown on the right. B: Across all simulations of the 200 motor neuron *m* = 2 model, the total strength of the connections from the reservoir to the agonist motor neurons divided by the total strength of the connections from the reservoir to the antagonist motor neurons. Bar height indicates the mean across the five simulations and the error bars represent 95% confidence intervals. C: Across all simulations of the 200 motor neuron *m* = 2 model, the standard deviation of the connection strengths from the reservoir to the motor neurons. Bar height indicates the mean standard deviation across the five simulations.

Fig 5A shows an example, from the first simulation run compared to its yoked control, of the learned synaptic weights from the reservoir output to the motor neurons. The figure illustrates how the salience-based reinforcement resulted in the connection weights to the agonist motor neurons being stronger than the connection weights to the antagonist motor neurons. Indeed, a paired sample t-test comparing the ratio of the mean of the connection weights to agonist motor neurons divided by the mean of the connection weights to the antagonist motor neurons showed this to be significantly higher for the salience-reinforced simulations (mean ratio of 1.35) than for their yoked control simulations (mean ratio of 1.00), *T*(4) = 18.11, *p* < .001 (Fig 5B). It seems likely that the greater connection weights to agonist motor neurons are responsible for the greater average activity of the masseter and orbicularis oris after salience-reinforced learning, in turn leading to more salient and more syllabic vocalizations.

As shown in Fig 5C, the standard deviations of the synaptic weights from the reservoir to the motor neurons is also greater for the salience-reinforced simulations (mean standard deviation of 0.61) compared to their yoked control simulations (mean standard deviation of 0.58), *T*(4) = 5.06, *p* = .007. Future work is needed to determine if difference increase in variability of weights is related to the simulations’ vocalization behaviors and if so, how.

## Discussion

### Summary

It is very difficult to measure infants’ neural activity in situ while they learn to babble. Computational modeling can help to identify plausible mechanisms underlying vocal development and evolution.

We presented a computational account of canonical babbling as a consequence of general purpose learning in the motor cortex. The model combines a spiking neural network model of motor cortex subpopulations with a simulation of the human vocal tract. The neurons in the neural network control the jaw (masseter) and lip (orbicularis oris) muscles in the vocal tract simulation, allowing for control of degree of mouth opening. The vocal tract simulation permits the synthesis of sounds. The model is reinforced for producing high salience sounds (relative to the sounds it has previously produced), assuming infants have greater interest in the more salient sounds that they produce and/or that more salient sounds are more likely to elicit adult attention and response. Both intrinsic interest and social response are assumed to activate reward centers in the infant nervous system. Reward is assumed to modulate spike timing dependent plasticity in the spiking neural network, allowing the model to learn to recreate some of the activity patterns that lead to vocalizations that received a reward.

Over time, the model learned to increase the salience of its vocalizations, which coincided with increase in the number of syllables it produced. As a result of this learning, at the end of the simulation the model showed increases in both the range of oscillation of jaw and lip muscle activity and in the baseline level of jaw and lip muscle activity, both of which were more conducive to producing movements that led to alternation between the jaw being open and the jaw being closed, which was associated with the production of consonants.

### Theoretical implications

The present study has implications for our understanding of how the nervous system supports the development of syllabic babbling in infancy as well as for how syllabic speech may have evolved. It demonstrates that the dynamics of activity of small groups of cortical neurons, when converted to muscle activity, are sufficient to generate oscillatory movement of vocal tract structures in such a way as to generate canonical syllables, as in human infants’ reduplicated canonical babbling. Not all simulated cortical activity will lead to consonant-vowel alternation; the degree of oscillation must be sufficiently great and the baseline level of activity must be at an appropriate level so that oscillation around that baseline will lead to alternation between mouth closure and mouth opening. These modifications to the cortical oscillation are what the model learns, and may be the primary things the human infant brain learns to accomplish over the course of the first year of life, as it gradually acquires the tendency to produce syllabic sounds and, once it can produce them, to produce them with increasing frequency.

Our preliminary simulations exploring the muscle scaling and number of output and motor neurons parameters indicated that it is possible to obtain high rates of syllable production without any learning, simply by setting both these parameters to high values. This raises the question of why, if it is so easy to generate syllabic sounds even without learning but simply by having a large degree of connectivity between cortex and vocal tract muscles, human infants do not babble from birth but instead appear to learn the behavior. Perhaps having high amounts of general body movement, including vocal tract structures, from birth would disadvantage the infant. In the absence of a good reason to move a body part, it may be best to keep it relatively inactive, both to save metabolic resources and for safety reasons (for example, high degrees of vocal tract movement in early infancy might interfere with swallowing and breathing). Starting from a default state of relative inactivity and then learning to gradually increase activity of specific muscles toward an adaptive aims (i.e. in ways that increase social or other rewards) may be a better evolutionary strategy.

The point of view supported by our model is consistent with Andrew’s [77] argument that cyclic patterns are characteristic of systems that have feedback control elements. Feedback control elements are certainly present in the cerebral cortex as well as in the spiking neural network used in our model. The individual neuron dynamics are subject to feedback control mechanisms and the reservoir model also has a balance of excitatory and inhibitory activity, along with recurrent connectivity, that lead to oscillatory behavior. An advantage of recurrent spiking neural network models is that they incorporate these different levels of feedback control, generating dynamics that have rich oscillatory activity as has been observed in measurements of electrical activity of cortical populations.

MacNeilage [15] proposed that the rhythmic jaw movements that appear at around 7 months of age are derived from central pattern generators (CPGs) responsible for evolutionarily older vocal tract functions, in particular chewing and lipsmacking. MacNeilage thought it implausible that “speech would develop an entirely new rhythm generator, with its own totally new superordinate control structures, which could respond to coordinative demands similar to those made on the older system, if evolution is correctly characterized as a tinkering operation, making conservative use of existing CPGs” (p. 503). It is certainly quite possible that subcortical circuitry is also involved in the generation and adaptation of rhythmic vocal tract movements associated with canonical babbling development [17, 25, 78–82]. Nevertheless, our model shows that it might not be so unreasonable an idea after all that new structures are recruited when an infant learns to produce canonical babbling. Since cortical networks readily generate oscillatory activity, perhaps once more direct cortical control over vocalization evolved, it became more straightforward for the cortex to generate rhythmic facial movements that are timed together with phonation at the larynx. Whether it is easier to evolve to repurpose existing ingestion and lipsmacking CPGs for speech-related babble sounds than to learn to harness cortical dynamics for directly controlling the vocal tract in a speech-like way is an empirical question. Computational models combined with neurophysiological measurements from cortical and subcortical structures alongside longitudinal and comparative behavioral measurements will help to identify which of these, or whether a combination of the two, is the most likely basis for emergence of syllabic speech.

While our model suggests that an evolutionary root in brainstem CPGs for chewing or lipsmacking may not be strictly necessary, it is quite possible that both brainstem and cortical programming of vocal tract movements may both contribute to speech movement. The most likely possibility may indeed be that the development of canonical babbling is so critical to modern human function that it has become canalized, supported by a robust combination of multiple neural and environmental processes [14, 26].

As discussed in the Introduction, the majority of the existing computational models to date have focused either on how infants acquire vowel categories or on how they acquire the consonant-vowel combinations that make up their language. The work on acquisition of consonant-vowel combinations has tended to assume that the agent already has a concept that speech is organized into syllables, and that the model’s task is to figure out how to combine articulator movements within this syllabic base, to produce combinations like [bab] or [mi] that have various combinations of consonants and vowels, forming syllables that could constitute words in the language. This is consistent with some work on human infant babbling, which has argued on the basis of phonetic transcription of infants’ canonical babble and first words that the most significant achievement in infant vocalization is the increased control over the movement of various vocal tract articulators, including the tongue and lips, superimposed on a frame of jaw movement [83]. In contrast to those previous computational modeling efforts, the present work provides an account for how the precursor to this learning of phonetic content of syllables, i.e. the syllabic jaw oscillation frame, might itself be learned. This may help account for the fact that canonical babbling does not appear consistently in infants’ repertoires until the second half of the first year as well as for why it appears to increase rather gradually in frequency over a period of several months. The present model is the first to attempt to provide a neurophysiological account for this earlier precursor phenomenon.

### Predictions

The model and general theoretical perspective we have presented here makes several predictions that can potentially be tested through future studies with human participants. Neurophysiologically, it predicts a correlation between the activity in motor cortex regions controlling the masseter and orbicularis oris and the production of bilabial canonical syllables in infants, both at immediate (millisecond/second) timescales and at longer (days/weeks/months) timescales, as infants acquire the ability to produce syllabic utterances. This could perhaps be tested using a methodology similar to that in [23]. It also predicts that higher concentrations of dopamine in motor cortex will be observed when infants listen to more salient sounds, such as syllabic speech, and/or when one of their vocalizations receives a positive response from an adult.

The work presented here also makes a number of predictions that could be tested using behavioral methods. One such prediction is that infants will prefer to listen to canonical babbling as opposed to non-canonical babbling (although if this is not the case, it is still possible that the mechanisms presented here could be operating with adult responses being the main source of reward). Whichever the source of reward, whether it be infants’ own sound preferences or adults’ contingent responses, our model predicts that as infant production abilities increase, i.e. as their vocalizations become more syllabic, the threshold for receiving a reward and for release of dopamine (or some other plasticity-modulating neurotransmitter) will increase. If it turns out that infants do prefer to listen to more salient, syllabic sounds, indicating that it is likely that self-stimulation is serving as a reinforcer, then we would expect that as infants’ produce syllabic sounds more frequently, stimuli with more consonants per unit time will be needed to elicit and maintain their interest. On the social reinforcement side, we expect parents’ responses to infant vocalizations to become more contingent on higher syllabicity of the infant vocalization as their infants’ vocalizations become more advanced. Finally, the model predicts that if positively reinforced through contingent responses in a laboratory setting specifically for producing syllabic sounds, infants’ subsequent vocalizations should become more syllabic, even when the acoustic content of the contingent response remains the same. This prediction could be tested through a straightforward modification to previous protocols for contingently reinforcing infant vocalizations in the lab [73], though it would require a reliable method for assessing syllabicity of infant vocalizations in real time.

### Limitations and future directions

As argued above, in its current form, the model demonstrates a number of features that already can help inform our understanding of how speech develops and how it may have evolved. However, a number of simplifications were made in order to make the model building and the analysis of its performance tractable. Additionally, there were a number of features of the model that were chosen or designed rather arbitrarily. The implications of these decisions, and the effect of making different choices, should also be addressed in future work. In this section, we highlight some limitations of the current model and discuss how addressing them could lead to interesting future directions.

One obvious simplification, mentioned in the previous section, was that the model had only one motor degree of freedom. Only jaw and lip muscles were controlled by the neural network, and they were controlled together, so that they were always activated in perfect synchrony with each other. In reality, these two structures can be controlled independently, and even have multiple independently controllable muscles affecting them. Furthermore, there are many other vocal tract structures whose muscular control was not learned by the model. These include the tongue, the velum, the pharynx, the larynx, and the lungs. Future work should have the model manipulate more or even all of these structures. Besides being a good test of whether the model can still learn under such conditions, this will potentially allow many more aspects of early vocal learning to be addressed. For example, if the larynx were manipulated, this would permit the modeling of cortical learning of laryngeal control, which also takes place over a protracted period of time during the first year and which is an important component of canonical babbling development, as without phonation, no canonical syllables can be heard no matter how the jaw, lips, or tongue are moving. Infants have been observed to produce silent jaw oscillations without phonation [84], perhaps reflecting that they are still in the process of exploring and learning about the relationship between the larynx and the jaw in sound production. Another worthwhile future direction would be to incorporate control of the various muscles of the tongue, in which case a good modeling target would be to simulate existing data on how tongue movements relate to activity in various cortical regions during speech [23] or to see if the neural model can account for the relative frequencies of different consonant-vowel combinations in infant babbling (e.g., along the lines of [85]).

Another simplification was that the the model did not contain a perceptual system. Acoustic analysis of vocal sounds in order to determine when rewards occur was done separately, through a statistical model that aims to mimic some features of humans’ neural processing of speech stimuli. Incorporating neurons into the model that perform perceptual processing of the sounds the model makes as well as of the sounds others in the environment produce would enable the model to address other phenomena related to infant vocalization. For example, it is known that the content of adults’ responses to infant vocalizations plays a role in shaping future infant vocalizations. Multiple labs have found that infants’ future vocalizations tend to take on phonetic characteristics that resemble those in the responses they previously received. In particular, when adult responses are verbal or syllabic, this tends to promote future infant vocalizations having syllabic properties as well, more than when adult responses are nonverbal or nonsyllabic [73, 86]. Future work should address this more complex issue of possible mechanisms whereby the perceptual content of adult vocalizations combines with the rewarding value of contingent responses to shape infant vocalization qualities (see [87] for a proposal on how some of this might be accomplished). It would also be helpful to be able to simultaneously model perceptual and motor speech learning, as there is evidence that learning in each of these modalities affects learning in the other [26, 88, 89].

Of course, there are many more additional extensions that could also prove useful in testing different hypotheses about how syllabic babbling develops. For example, human infants exhibit many other types of rhythmic motor activities during the first year [18]. In fact, the development of canonical babbling appears to be predicted by the co-occurrence of rhythmic limb movements with vocalization, suggesting the possibility that rhythmic movement of other effectors influences babbling development or at least that the two are related by a common underlying mechanism [90–92]. The influence of rhythmically oscillating neural circuits from another modality on the neural circuits controlling the vocal tract could potentially be explored with a model based on the one presented here.

Eventually it would be good to make comparisons to human data in a more detailed way than has been done here so far. If the model could be made, perhaps through some of the future directions suggested above, to vocalize not every second but at a rate that better matches infants’ actual volubility, and if social reinforcement patterns could be made, by reference to human data, to more accurately match the rates and temporal patterns of actual social reinforcement (see [93, 94] for some ideas on how this might be done), and if more detailed information on the frequency and temporal patterning of bouts of canonical babbling production were available, it would be possible to compare the model’s canonical babble production to the rates and temporal patterning of canonical babble production produced by human infants. It is likely that the model would need further modifications to provide a good fit, so the exercise would help to build a more realistic model.

There are also a number of variations on the neural network architecture and learning rules that would be worth exploring. Indeed, a number of modifications might be necessary in order to perform some of the future directions just discussed. The number of subgroups of neurons, the degree of connectivity between neurons, the ways in which synaptic connectivity is scaled, and so on are all parameters that can be explored to see how they affect neural and motor dynamics as well as learning patterns [95, 96]. Using such a simple reservoir architecture also has limitations in terms of neural plausibility. The neural subgroups, connectivity, etc. could be modified to provide a fit with what is known about neural circuitry in the human (ideally the human infant) nervous system, and updated as new information becomes available. Guenther and colleagues [52] have provided a particularly compelling example of how subregions of the central nervous system can be incorporated into a (non-spiking) neural model of speech production, with model features and findings mapping directly to neuroimaging data. In particular, as more articulators are added, it will likely be important to increase the neural network size, and partitioning it into subgroups or modifying the connectivity to have less uniform and more realistic connectivity statistics may be important both for biological realism and for performance. Some other features worth exploring are rewards that vary in degree (i.e. rewards that are not binary on or off) [97] and the manner in which neural activity is smoothed or filtered prior to using it to control muscle activity. Additionally, learning not only in the connections between the reservoir and motor neurons but also within the reservoir itself should also be explored.

Finally, in the present study, we used an adult female vocal tract model, simulated on a computer and unchanging over time. It would be worth exploring other vocal tract modeling approaches, in particular using simulated vocal tracts with shapes and other features (mass, elasticity, etc.) matched more closely to infant physiology [98–101]. This would allow the physiology of the infant to be more accurately taken into account and would make it possible to ask how changes in vocal tract physiology during the first year might facilitate or interfere with learning to produce syllabic sounds. Additionally, headway is being made into creating a robotic model of an infant vocal tract [102]. Such an approach has the advantage of not relying on simulation assumptions to generate acoustics. A robotics approach also may speed up the simulation time and make real-time interaction with the model more feasible (the first-principles simulation of vocal tract mechanics and acoustics is currently the most computationally intensive portion of our model). Current challenges include material cost and high time to development compared to using an off-the-shelf vocal tract simulator.

Despite the simplifications made in the model presented here, our results provide good reason to believe that cortical dynamics and learning may underlie the development of syllabic vocal behavior. The work demonstrates how an approach combining spiking neural network modeling and vocal tract simulation can be used to model potential scenarios for how syllabic vocal abilities are learned, providing impetus for pursuing these various future directions.

## Supporting Information

S1 Sound Example of a vocalization containing no consonants. (WAV)

S2 Sound Example of a vocalization containing one consonant. (WAV)

S3 Sound Example of a vocalization containing multiple consonants. (WAV)

S4 Sound Samples of the vocalizations produced by the first run of the salience-reinforced model over the course of learning. (WAV)

S5 Sound Samples of the vocalizations produced by the second run of the salience-reinforced model over the course of learning. (WAV)

S6 Sound Samples of the vocalizations produced by the third run of the salience-reinforced model over the course of learning. (WAV)

S7 Sound Samples of the vocalizations produced by the fourth run of the salience-reinforced model over the course of learning. (WAV)

S8 Sound Samples of the vocalizations produced by the fifth run of the salience-reinforced model over the course of learning. (WAV)

S9 Sound Samples of the vocalizations produced by the first yoked control run over the course of learning. (WAV)

S10 Sound Samples of the vocalizations produced by the second yoked control run over the course of learning. (WAV)

S11 Sound Samples of the vocalizations produced by the third yoked control run over the course of learning. (WAV)

S12 Sound Samples of the vocalizations produced by the fourth yoked control run over the course of learning. (WAV)

S13 Sound Samples of the vocalizations produced by the fifth yoked control run over the course of learning. (WAV)
